# Living with idiopathic basal ganglia calcification 3: a qualitative study describing the lives and illness of people diagnosed with a rare neurological disease

**DOI:** 10.1186/s40064-016-3390-z

**Published:** 2016-10-04

**Authors:** Tomiko Takeuchi, Koko Muraoka, Megumi Yamada, Yuri Nishio, Isao Hozumi

**Affiliations:** 1 Department of Gerontological Nursing, Toyama University, Toyama, Japan; 2 Department of Adult Health Nursing, Toho University, Tokyo, Japan; 3 Department of Neurology and Geriatrics, Gifu University, Gifu, Japan; 4 Laboratory of English Studies, Gifu Pharmaceutical University, Gifu, Japan; 5 Laboratory of Medical Therapeutics and Molecular Therapeutics, Gifu Pharmaceutical University, Gifu, Japan

**Keywords:** Idiopathic basal ganglia calcification (IBGC), Primary familial brain calcification (PFBC), Fahr disease, Narrative, Qualitative research

## Abstract

**Purpose:**

Idiopathic basal ganglia calcification (IBGC) is a rare, intractable disease with unknown etiology. IBGC3 is a familial genetic disease defined by genetic mutations in the major causative gene (*SLC20A2*). People with IBGC3 experience distress from the uncommon nature of their illness and uncertainty about treatment and prognoses. The present study aimed to describe the lives and illness of people with IBGC3.

**Methods:**

Participants were recruited from patients aged 20 years or older enrolled in a genetic study, who were diagnosed with IBGC3 and wanted to share their experiences. In-depth semi-structured interviews were conducted with six participants. Interviews were conducted between December 2012 and February 2014, and were recorded and transcribed verbatim. Qualitative data analysis was performed to identify categories and subcategories. Efforts were made to ensure the credibility, transferability, dependability, conformability, and validity of the data.

**Results:**

Six thematic categories, 17 subcategories, and 143 codes emerged. The six categories were: (1) Frustration and anxiety with progression of symptoms without a diagnosis; (2) Confusion about diagnosis with an unfamiliar disease; (3) Emotional distress caused by a genetic disease; (4) Passive attitude toward life, being extra careful; (5) Taking charge of life, becoming active and engaged; and (6) Requests for healthcare.

**Conclusions:**

The qualitative data analysis indicated a need for genetic counseling, access to disease information, establishment of peer and family support systems, mental health services, and improvement in early intervention and treatment for the disease.

## Background

Idiopathic basal ganglia calcification (IBGC), also called Fahr disease, is a rare and intractable disease. It shows abnormal deposits of calcium in the basal ganglia, the dentate nuclei of the cerebellum, the pulvinar thalami, and subcortical white matter (Manyam 2005). For a diagnosis of IBGC, other known causes of calcification, including metabolic, infectious, toxic, or traumatic causes should be excluded (Bonazza et al. 2011). The clinical features of IBGC include parkinsonism, cerebellar symptoms, cognitive impairment, psychosis, seizures, and chronic headache. Patients may also be asymptomatic.

Historically, more than 37 names had been used to describe the disease. Recently the designation “primary familial brain calcification (PFBC)” has been proposed in opposition to ‘secondary’ and on the basis of family history (Sobrido et al. 2014). The designation and classification from the genetic background are needed for further study to clarify the pathophysiology. With vague criteria and an unknown etiology, Fahr disease was a blind spot in medical care. The discovery of the mutations in the gene *SLC20A2* which cause IBGC3 was a turning point in understanding the disease’s pathophysiology. Recently, other causative genes have been reported, especially in familial IBGC (FIBGC) cases, and classified as IBGC 3–6 (Wang et al. 2012; Yamada et al. 2014; Nicolas et al. 2013; Keller et al. 2013: Legati et al. 2015). The phenotypes are thought to not necessarily be correlated with the genotypes (Nicolas et al. 2015). In most patients, FIBGCs are considered to be autosomal dominantly inherited.

Even today, IBGC itself is not well known to the general public. There is also insufficient information available via social networking or similar agencies to further understanding of the disease. Therefore, despite a diagnosis and some medical information provided by their doctors, people with a diagnosis of IBGC may be upset by an unfamiliar genetic disease, and experience despair. To date, no clinical research on the mental health needs of people with IBGC has been available. FIBGC type 3 is the most common type of FIBGC worldwide, including in Japan (50 % of all cases) (Yamada et al. 2014). Therefore, this study was conducted to allow definitive examination of a homogeneous sample of patients with IBGC3, and provide an initial profile of their self-reported social, medical, and psychological challenges. Patients with IBGC3 were interviewed after informed consent was obtained. We conducted a qualitative analysis of the interview data, and sought to better define the medical support needs of patients with IBGC3.

## Methods

### Participants

In our ongoing genetic study, ten patients had been diagnosed with IBGC3. Participants were recruited from those patients who were over 20 years of age and wanted to talk about their experiences. Six patients participated in the present study, two females (F1 and F2) and four males (M1–4). Clinical features of the participating patients are shown in Table 1.

**Table 1:** Clinical features of study participants (n = 6)

	F 1	F 2	M 1	M 2	M 3	M 4
Mutation	c.344C>T	c.1909A>C	c.344C>T	c.1909A>C	c.1399C>T	c.1848G>A
	T115M	S637R	T115M	S637R	R467X	T616X
Participant information						
Place of residence	Hokkaido island	The main island	Hokkaido island	The main island	Shikoku island	Shanghai in China
Age at onset (years)	58	60	46	Unknown	15	13
Age at detection of calcification (years)	60	60	50	22	23	25
Age at diagnosis of IBGC3 (years)	69	64	50	22	26	26
Onset symptom	Dysarthria	Dysarthria	Easy to anger	–	PKC	PKC
Interview						
Age at interview (years)	72	66	50	22	26	28
Symptoms at interview	Forgetful, headache	Speech, gait, forgetful, headache	Headache, cramp, forgetful	Unstable	Cramp	Headache, unstable
Family information						
Other patients in the family	Her son and five relatives	Her son	His mother and five relatives	His mother	His mother	Three relatives
References	II-1 in F3 Case 3, Yamada et al. (2014)	III-1 in F1 Case 1, Yamada et al. (2014)	II-1 in F3, Yamada et al. (2014)	II-2 in F1, Yamada et al. (2014)	Case 5, Yamada et al. (2014)	Figure 1

The genetic mutations of five patients (T115M, S637R, T115M, S637R and R467S) have been identified in a previous paper (Griffiths et al. 2007). In addition to the data reported in the previous paper (Griffiths et al. 2007), the genetic mutation of M1 was confirmed to be identical to that of his mother (F1). At the time of interview, participants’ ages ranged from the late 20s to early 70s. The interval since being informed of their diagnosis varied from a few months to 3 years. M1 and M2 are sons of F1 and F2, respectively. M1, M2, and M3 were not married. All participants except M2 had symptoms. Although M2 felt well, his mother (F2) recommended he go to a hospital.

**Fig. 1:**
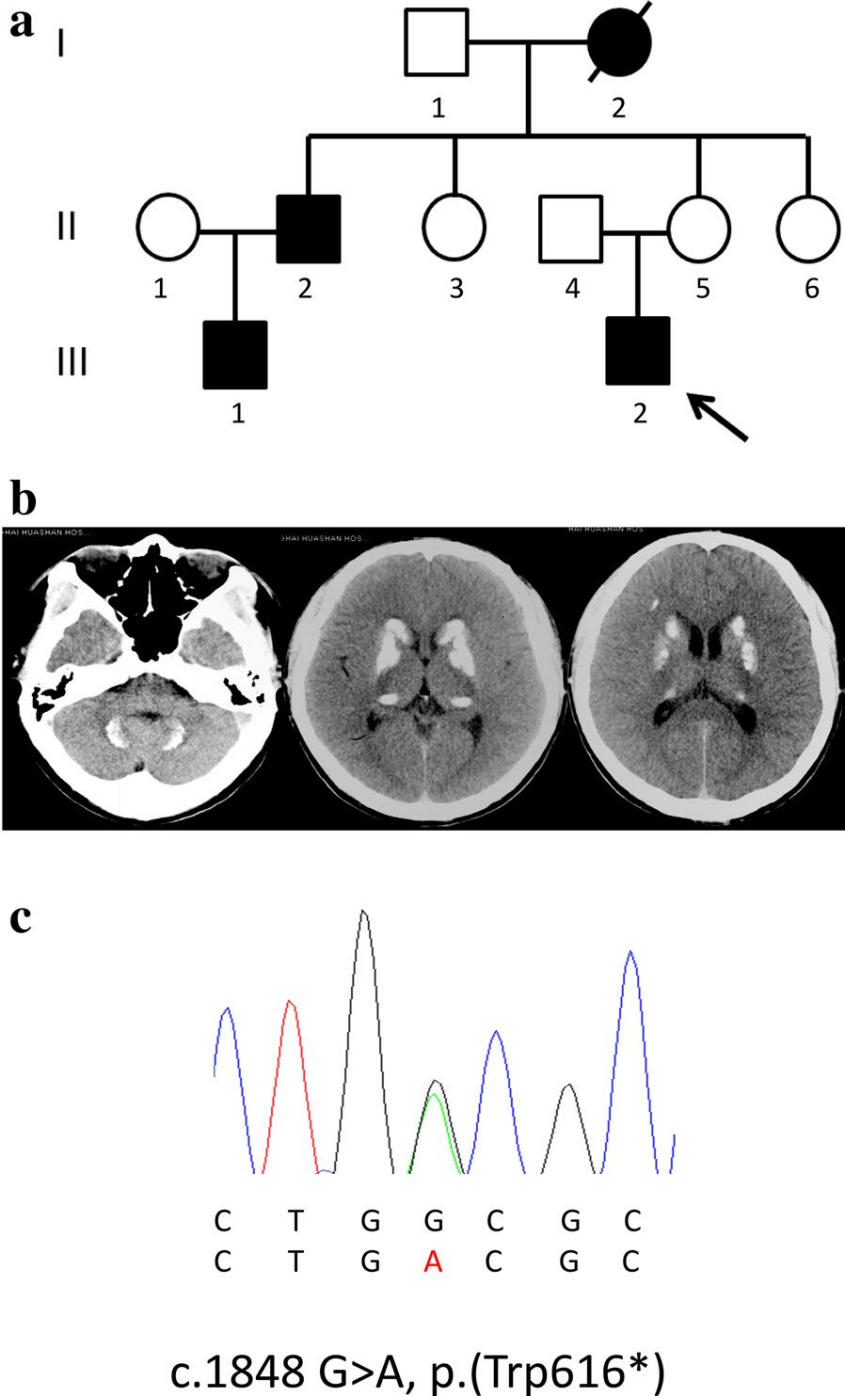
**a** Family tree of Case M4. The *arrow* indicates the index subject. *Filled symbols* represent family members affected by calcification. **b** Computed tomography images of Case M4. Typical calcification was seen in bilateral basal ganglia, dentate nuclei in the cerebellum, and the subcortical white matter. **c** DNA sequence electropherogram showing the mutations [c.1848 G>A, p.(Trp616*), inducing a stop codon in exon 11] in the patient (M4). *Letters in red* indicate mutated sequences

A new nonsense mutation, c.1848 G>A, p.(Trp616*), inducing a stop codon in exon 11 of *SLC20A2* and resulting in a putative truncated protein, was discovered in M4 (III-2, in Fig. 1a), a 29-year-old man. He had suffered from paroxysmal kinesigenic choreoathetosis (PKC) and had taken carbamazepine since age 13 years. His brain computed tomography (CT) images revealed a typical pattern of calcification in the bilateral globus pallidus, caudate nuclei, putamen, pulvinar thalami, dentate nuclei, and subcortical white matter (Fig. 1b). He obtained information about his and his family members’ diseases from a doctor in China: his uncle (II-2), his cousin (III-1), and his grandfather (I-2) had all suffered from calcification in the brain (Fig. 1c), but the mutations causing PKC had not been found in his genetic analysis. He was taken to the present authors’ hospital by his Japanese wife for a medical examination. The medical examination revealed that the PKC was well controlled, but he experienced headaches when he had a cold.

### Interview records

Participant interviews were conducted between December 2012 and February 2014. Interviews were performed in a quiet, closed room in a hospital or hotel, with no other person present. Participants were interviewed once, with interviews lasting 50–90 min. The six semi-structured interviews were based on a loose structure comprising open-ended questions, and were conducted according to the methodological orientation of Sandelowski (2000). Interviews were face-to-face and performed by a professional interviewer who first introduced herself and obtained written informed consent.

Each interview was recorded with a digital voice recorder. The protection of participants’ privacy was paramount. The interviewer encouraged participants to speak about their experiences as freely as possible. The interview questions were: “How did you find out about your disease?”; “What did you think when you heard the diagnosis of your disease?”; “How do you feel about your disease at present?”; “How do you feel about your family after hearing your diagnosis?”; “Is there anything you have learned through your experiences?”; and “What do you expect in terms of medications and medical care?”

### Analysis

All statements made by participants in the interviews were recorded and fully transcribed. The credibility and transferability of interview texts were checked by two researchers. The transcribed texts were analyzed using qualitative data analysis software, MAXQDA10 (VERBI Software, Berlin, Germany). First, all data were carefully interpreted and the transcriptions divided into partitions according to content. Each chunk was coded with definite terms using the words from the participant’s narrative. In this procedure, chunks were rearranged over the time course. Next, codes were summarized by a suitable concept and named with representative terms (subcategorization). All data were examined again carefully and the content of codes and subcategories were re-examined. Finally the subcategories were summarized in wider concepts and named according to key characteristics (categorization). To ensure the validity of the data interpretation, the results were checked by the participants and other members of the research team.

## Results

Six categories, 17 subcategories, and 143 codes were extracted: categories are shown using () and subcategories are shown by ≪≫ (Table 2). In terms of coding the six categories, all participants reported similar experiences and impressions. For example, a participant with some symptoms had consulted a doctor, but felt more anxious about the disease progression because the doctor did not provide a proper diagnosis. When the participant was finally informed of his diagnosis with an unfamiliar disease, he was upset. In addition, he felt distressed by hearing the disease was a genetic disease and feared personality changes due to cryptogenic abnormalities. He also wished for a better medical system and the opportunity to ask questions of medical doctors.

**Table 2:** The coding tree

Categories	Subcategories
Frustration and anxiety with progression of symptoms without being diagnosed	Had not felt any physical abnormality in their teens Were recommended to consult doctors by their friends and parents Had obsessed with anxiety about the unknown disease and progression of their symptoms
Confusion about diagnosis with an unfamiliar disease	Got upset when they heard the diagnosis was a genetic and intractable disease Calmly understood the situation afterwards Did not check because there was fear about knowing Were not able to obtain sufficient information about the disease on the Internet and were not satisfied with the information
Emotional distress caused by a genetic disease	Tended to avoid contacting their brothers and sisters, who did not have the same symptoms Confessing to their supervisors about contracting the disease to account for their absences to consult doctors Concerns for marriage and childbirth
Passive attitude toward life, being extra careful	Dealt with things while embracing the anxiety Had experienced troubles in life that others could not know
Taking charge of life, becoming active and engaged	Were willing to communicate with other patients with the same disease Recognized that life was not only for oneself
Requests for healthcare	Specialized doctors and hospitals should be increased medical financial supports must be provided Expected enactment of laws allowing euthanasia

The six categories were: (frustration and anxiety with progression of symptoms without being diagnosed), (confusion about diagnosis with an unfamiliar disease), (emotional distress caused by a genetic disease), (passive attitude toward life, being extra careful), (taking charge of life, becoming active and engaged), and (requests for healthcare).

In addition, we reported participants’ “questions for medical doctors”. Each category and corresponding subcategories are discussed below (Table 2).

### Category 1: (frustration and anxiety with progression of symptoms without being diagnosed)

Two patients, M3 and M4, who had shown symptoms of PKC reported that they ≪had not felt any physical abnormality in their teens≫. However, in their 20s, they ≪were recommended to consult doctors by their friends and parents≫ because of gait disturbance and toppling falls. Other participants, F1 and F2, were also recommended by their husbands and daughters to consult doctors because of forgetfulness and articulation disorders. F1 and F2 had suffered from the disease for 2–13 years, and reported they ≪had obsessed with anxiety about the unknown disease and progression of their symptoms≫.

*“I was told ‘you are limping’ by my colleagues in my work place. I knew that others had recognized my disability. I tried to repeat the action of ‘stop and move’ but I felt paralysis about once per 100 times. Before that I had not paid attention to it, but I decided to consult an orthopedist.”* (M3)

### Category 2: (confusion about diagnosis with an unfamiliar disease)

Participants M4 and F2 had taken proactive action to seek a clear diagnosis. F2 was recommended to consult a doctor at a university hospital by an acquaintance with similar symptoms. M4 had obtained information on the disease via the Internet and consulted a medical doctor specializing in neurology at Gifu University Hospital. They had been examined and informed of their IBGC3 diagnosis. Both M4 and F2 mentioned they felt fear about the progression of the disease and ≪got upset when they heard the diagnosis was a genetic and intractable disease≫. M2, who was thought to be presymptomatic ≪calmly understood the situation afterwards≫. M2 (asymptomatic) was the only participant who ≪did not check because there was fear about knowing≫. The other male participants, M1, M3, and M4, were confused on hearing their diagnosis and reported that they ≪were not able to obtain sufficient information about the disease on the Internet and were not satisfied with the information≫, in which the symptoms varied widely.

*“Before I was told the name of the disease, I had been told I suffered from a genetic intractable disease. I was shocked and my head went blank. I knew the name ‘Fahr disease’ when I saw the name on the examination paper written by another doctor.”* (F2)

*“At that time I was a little bit shocked. I was really shocked. Because I knew that disease. I knew how it is going to be…But I have to shout.”* (Spoken in English by M4)

### Category 3: (emotional distress caused by a genetic disease)

Four younger patients, M1, M2, M3, and M4, experienced great distress when they realized the disease was a genetic disease. M1, M2, and M3 reported that they ≪tended to avoid contacting their brothers and sisters, who did not have the same symptoms≫. M2 and M3 had tried to cover up the situation with their work colleagues, although they ended up ≪confessing to their supervisors about contracting the disease to account for their absences to consult doctors≫.

M3, who had a fiancée, and M4, who was married, reported having ≪concerns for marriage and childbirth≫. M3 mentioned that his fiancée understood his situation and disease, but her parents and relatives were strongly opposed to their marriage. They had seriously considered the possibility of adopting children.

*“I started to worry about the future. Maybe we are going have kids, what if, what if going to have a kid…Ah, yes. I changed to a healthy life style. I’m having healthy diet…I had talked with my wife a couple of times during this half year. From my side, I think…I’d still like to, to have a child, somehow.”* (Spoken in English by M4)

### Category 4: (passive attitude toward life, being extra careful)

Symptoms of IBGC such as forgetfulness, headache, and clumsiness were not easily recognized without the participants’ acknowledging them, even by their families. Participants tended to hide their symptoms and were careful not to make mistakes or cause trouble for others. They did not show their disabilities caused by the disease. This made participants more introverted and reserved. F2 tended to drive the car on the center of the road. M1 could not write letters when he was tired. M4 and M1 tended to have passive attitudes because of their fear of what may happen.

*“Somehow I worry about the disease when I drive a car. When my car was driven in the center of the road, I felt there was something wrong with me…I’ve been be very careful in driving.”* (F2)

*“I have stones in my brain, haven’t I?…just like stones in tofu (soybean curd). If I shake my head strongly, moving stones would damage the tofu…Then I always try not to shake my head in my life.”* (M4)

Most participants were concerned about the disease and tried to manage the situation. F1 mentioned that she ≪dealt with things while embracing the anxiety≫. Participants reported they ≪had experienced troubles in life that others could not know≫. M2 remarked he tried to forget the disease. In contrast, some participants tried to behave proactively. M2 and M4 were willing to have regular check-ups. M3 wanted to buy medical insurance before onset of the disease.

### Category 5: (taking charge of life, becoming active and engaged)

Participants ≪were willing to communicate with other patients with the same disease≫ and get useful information from others. They felt frustrated with the lack of medical information. F2, M1, M2, M3, and M4 were willing to take a part in a community for patients with IBGC. M1 also wanted to get into an Internet communication forum. In contrast, F1 who had taken a part in a community for patients once before, was not interested in participating again because she found that others only talked about their concerns. M2 and M3 had mixed feelings: they wanted to talk about their situation with others, but not those with more severe symptoms.

“*I think that the symptoms vary widely. Perhaps my symptoms seem to be mild. If other patients have more severe symptoms than mine, I’m afraid that it would hurt others’ feelings. This means that I would have to watch what I say.”* (M3)

Some participants changed their perspective on their lives after being diagnosed with a rare, intractable disease. M4 mentioned he ≪recognized that his life was not only for himself≫ and began to pay more attention to his health to prevent the disease progression, not only for himself but also for his wife. M2 and M3 understood that the disease had derived from their mother but did not blame anyone for the disease.

### Category 6: (requests for healthcare)

Some patients mentioned a desire for an improved medical system. F2, M3, and M4 felt that ≪specialized doctors and hospitals to deal with IBGC should be increased≫ because they had experienced several incorrect diagnoses from unspecialized doctors. All participants insisted ≪medical financial support must be provided≫. For example, F2 had finally quit the job she had been reemployed in when she was injured by a toppling fall, and M1 was excluded from night shifts because of less achievement in his job. In addition, M1, who had large calcification on his CT images, ≪expected enactment of laws allowing patients to practice euthanasia in Japan≫.

Participants were also asked whether they had some questions for doctors. Table 3 presents a list of these questions. In particular, participants wanted to know the relationship between headaches and the disease, and about calcification of the blood vessels and in the brain.

**Table 3:** Participants’ questions for medical doctors

What is the incidence rate of the disease?
Has the disease existed for a long time or is it a modern disease?
What is the most common symptom?
Is the occipital headache derived from the disease?
Do some people with calcification in the brain live without symptoms?
Is the intracranial pressure increased with the enlargement of calcification?
Is the brain damaged with strong movement because the brain is soft and calcification is hard?
Can thrombosis occur because calcification is seen in the blood vessels?
What is the proper time interval for the medical consultation?
How much does it cost for the full medical examination?
How is research for treatment progressing?

## Discussion

Qualitative research involving patients’ perspectives of their illness can generate new insights for medication, mental health care, and nursing, even when the number of patients is relatively small (Sandelowski 2000). Qualitative studies have been performed for major diseases such as cancer (Ziebland and McPherson 2006), rheumatoid arthritis (Hilton et al. 2009), and motor neuron disease (Newman et al. 2009; Locock et al. 2009; Caputo 2014). However, relatively few quantitative studies have been conducted on rare diseases, especially cancers (Griffiths et al. 2007; Witham et al. 2008), and there is little research on rare neurological diseases (Jaeger et al. 2015; Purcell et al. 2015). In Huntington’s disease, for example, patients’ quality of life, and the experiences and perspectives of family carers have been studied (Aubeeluck and Buchanan 2007; Williams et al. 2009; LoGuidice and Hassett 2005).

To date, 230 patients with IBGC in Japan have been enrolled in our genetic study. Although the etiology of IBGC was previously unknown, the mutations of *SLC20A2* encoding type III sodium-dependent phosphate transporter type III (PiT)-2 were reported in 2012 by Chinese researchers (Wang et al. 2012). Thereafter, several groups worldwide (including ours) have demonstrated that the mutation of *SLC20A2* is a major cause of familial IBGC (Wang et al. 2012; Yamada et al. 2014; Hsu et al. 2013). As IBGC3 (*SLC20A2*) is a genetically definite category and central to the syndrome, the results of clinical studies on IBGC3 are important and may be applied well beyond the confines of the present study to other types of IBGC. This study revealed three key points.

First, the qualitative analysis of the patients’ narratives clarified their worries and attitudes toward their lives. They had similar thought processes, including anxiety about the clinical features and future progress of the disease, particularly about observing their mothers and relatives, after they were told that the disease was familial and hereditary. They worried about marriage and childbirth and were anxious about needing full care in the future. Their attitudes had become introverted and focused on keeping their good health and not causing trouble to surrounding people.

Second, their questions and worries about the disease changed throughout their experiences in dealing with the disease. In the early stages, they could not get enough information through books or the Internet to understand the disease, and wanted to obtain information directly from medical doctors. However, they were told they suffered from a disease with an unfamiliar name and were at a loss for adequate information. For example, one participant (M1) thought he should not shake his head. The more correct information participants received, the more positive their attitudes were. Gradually, they were able to change their attitudes about their disease to be more positive.

Third, this study highlighted the psychological distress participants experienced because there is no medical cure for the disease. As they had been told that the disease was intractable, participants tended not to think about the disease. This coping method was similar to the behavior of patients with neurological intractable diseases reported by Higaki and Suzuki (2002). In addition, one participant wanted the option of euthanasia because the disease was not curable and he felt that death was imminent. Although he wanted to live a significant life, he experienced agony about his death.

Throughout the reported experiences of living with a familial intractable disease, we found some participants changed their minds about their lifestyles. They realized that their lives were not only for themselves, and would undergo regular check-ups and pay more attention to their health for their spouses and families. Kukinaka et al. (2013) reported similar feelings in patients with familial amyloid polyneuropathy.

Finally, this study has a key limitation. IBGC is a rare disease and the number of participants was therefore small. Generally, most patients with IBGC are sporadic and heterogeneous. We focused on genetically identified homogenous IBGC3. Although our conclusions are not meant to apply for all patients, those will be a foundation for the care of patients with IBGC or rare neurological diseases.

### Proposals for medical care for patients with IBGC3

Based on the present analysis, we identified three medical care proposals for patients with IBGC3.

#### Genetic counseling and mental health support

A genetic counseling and mental health support system should be established. Most patients with IBGC are worried about their genetic disease. According to the guideline for Prion disease in Japan, it is important for medical staff to understand patients’ and their families’ feelings, including anger, sadness, agony, and anxiety. Patients with IBGC and their families need support from medical staff to overcome psychological difficulties. We propose that medical staff consider the psychological processes of patients’ and their families’ and accept their feelings. This may help patients and their families to develop their ability to control and manage their feelings.

In addition, medical staff should identify methods to reduce patients’ anxiety about incurable diseases, to help prevent patients obsessing over the disease. Medical staff could educate patients about methods to decrease anxiety. Monat and Lazarus (1991) reported that a combination of direct medical treatments and psychological and emotional treatments is more effective than one treatment alone. Medical staff should therefore support patients both practically and psychologically.

In further discussion, the organization of genetic counselling deeply depends on how the health system is organized, reimbursed and how clinical genetics is integrated.

#### Questions and answers for medical information and early intervention

From a patient’s perspective, the most important considerations for medical staff are to prevent misunderstandings about the disease because of insufficient information, and provide adequate answers to their questions. “Q and A” handouts for patients should be updated regularly. In the early stages, medical staff should offer regular check-ups and instructions to patients; this is particularly recommended to improve the medical care for patients with IBGC3.

#### Patient support

The participants in our study tended to associate with other patients. Hotta and Hozumi (2012) reported that counseling and supporting systems were most desired by solitary (non-familial) patients with IBGC. Unlike other diseases, patients with rare and intractable diseases may be widespread and find it difficult to get together. In this case, medical staff should establish a patient association meeting or similar organized community, and perhaps use online platforms such as social networking to provide opportunities for patients to share information, anxiety, and feelings. Medical staff should observe the transitions of patients’ symptoms and feelings, support information exchanges to reduce feelings of isolation among patients with the same disease, and promote any necessary rearrangement of their life plans.

## Conclusion

This qualitative analysis of the narratives of patients with IBGC3 clarified their worries and attitudes towards their lives, the psychological changes that accompanied the progression of the disease, and their psychological distress as a result of insufficient medical information. We found that some patients were able to positively change their minds about living. This study also indicated a specific direction for the future medical care of patients with IBGC3. Patients highlighted the need for genetic counseling, mental health support, medical information “Q and A,” early intervention, and a patient community.

## Abbreviations

IBGC: idiopathic basal ganglia calcification
PFBC: primary familial brain calcification
PiT-2: type III sodium-dependent phosphate transporter
